# FDM 3D Printing of Flame-Retardant Polylactic-Acid-Based Composites with Electromagnetic Interference Shielding Performance

**DOI:** 10.3390/polym18182221

**Published:** 2026-09-12

**Authors:** Jun Li, Zijie Xu, Qinniu Lv, Xin Wang, Yinghong Chen

**Affiliations:** 1 State Key Laboratory of Advanced Polymer Materials, Polymer Research Institute of Sichuan University, Chengdu 610065, China; lj03040010@163.com (J.L.); xuzijie0310@163.com (Z.X.); lvqinniu_scu@163.com (Q.L.); 2 Research Center for Composite Materials, State Key Laboratory of Advanced Refractories, Shanghai University, Shanghai 200444, China

**Keywords:** fused deposition modeling (FDM), 3D printing, polylactic acid (PLA), flame retardancy, electromagnetic interference (EMI) shielding

## Abstract

In this work, flame-retardant (FR) PLA-based composites exhibiting both good flame retardancy and electromagnetic interference (EMI) shielding were fabricated through combining melt blending with fused deposition modeling (FDM) 3D printing. Intumescent flame-retardant (IFR) fillers and graphene nanoplatelets (GNPs) were incorporated to construct a synergistic system, and the resulting structure–property relationships were systematically investigated. The optimal performance was achieved with 15 wt% IFR fillers and 3 wt% GNPs. The prepared composite exhibited a residual char yield of 14.3% at 700 °C, significantly higher than that of neat PLA. Regarding flame retardancy, the limiting oxygen index (LOI) increased to 32.5%, and the vertical burning level achieved a UL-94 V-0 rating. In the cone calorimetry test, both the peak heat release rate and total heat release were markedly reduced, attributed to the formation of a dense and stable char layer. For EMI shielding, the FR composite achieved a total shielding effectiveness (SE_T_) of 20.6 dB in the X-band, with an absorption proportion exceeding 80%, indicating an absorption-dominated mechanism. Meanwhile, a well-established conductive network effectively enhanced the electromagnetic loss. Overall, the synergistic effect of IFR fillers and GNPs enabled simultaneous improvements in flame retardancy and EMI shielding in FR PLA-based composites, providing a feasible strategy for designing functional 3D-printed FR PLA-based materials.

## 1. Introduction

Among extrusion-based additive manufacturing (also named 3D printing) techniques, fused deposition modeling (FDM) has been widely adopted for fabrication of geometrically complex polymer components, although its performance strongly depends on the availability of suitable printable materials [1]. Polylactic acid (PLA) is particularly suitable for FDM due to its good biodegradability, melt processability, and printability [2,3]. However, the inherent flammability of PLA, reflected by its low LOI of approximately 20.5%, limits its use in applications where enhanced fire safety is required [4,5].

The increasing integration of electronic and communication devices has also intensified concerns regarding electromagnetic interference (EMI), particularly in 5G and Internet of Things (IoT) applications. This has created increasing interest in lightweight polymer composites capable of simultaneously providing EMI shielding and flame-retarding functions [6,7,8]. However, a single-filler system is often unable to simultaneously satisfy both flame retardancy and EMI shielding requirements. Conventional flame retardants mainly address fire safety, whereas conductive fillers such as graphene nanoplatelets (GNPs) are primarily introduced to establish conductive pathways for EMI shielding; neither strategy alone can readily provide balanced functionality and performance [9,10].

Previous studies have demonstrated that graphene-based nanofillers are effective in improving the EMI shielding performance and electrical conductivity of PLA-based composites through the construction of conductive networks [11,12]. However, PLA/GNPs nanocomposites have mainly focused on enhancing electromagnetic attenuation, while the inherent flammability of PLA has received comparatively less attention. Moreover, increasing the graphene content to achieve higher EMI shielding effectiveness may negatively affect the melt processability and printability of FDM filaments. Therefore, simultaneously achieving good flame retardancy, efficient EMI shielding, and favorable FDM processability remains challenging.

In recent years, researchers have attempted to incorporate intumescent flame retardants (IFRs) and GNPs into PLA matrices to achieve integrated flame-retarding and EMI shielding functions through a synergistic “char-forming-conductive” design strategy [13,14]. The interlayer interfaces and local pores generated during FDM printing may provide additional reflection and scattering pathways for electromagnetic waves and thus contribute to their attenuation [11,15]. At the same time, the nanoscale framework provided by GNPs can reinforce the IFR-derived char structure, thereby improving its compactness and thermal stability. As a result, the corresponding heat and smoke release could be reduced during combustion [16,17].

However, studies on FDM-printed PLA/IFR/GNPs ternary systems are limited. In particular, systematic investigations of the effects of filler composition and printing parameters on conductive percolation behavior, microstructure, and synergistic mechanisms are still insufficient. Moreover, the interplay between flame retardancy and EMI shielding has not yet been fully elucidated [18,19]. Although extensive studies have been reported on the flame-retardant modification and EMI shielding of PLA, the development of integrated multifunctional materials by simultaneously incorporating IFRs and GNPs into PLA and fabricating them via FDM 3D printing is still in its early stages [19,20,21]. The existing studies have mainly focused on optimizing individual properties, whereas there is lack of in-depth investigations into the synergistic effects between flame retardancy and EMI shielding [16]. In particular, the influence of FDM-induced interlayer features on the conductive network and the resulting multifunctional performance remains insufficiently understood.

Furthermore, the incorporation of flame-retardant fillers generally reduces the percolation efficiency of conductive fillers, whereas conductive fillers may in turn compromise the integrity of the intumescent char layer [22,23]. Therefore, achieving positive synergy between flame-retardant and conductive properties through the optimization of material composition and processing conditions presents a key challenge in this field.

To address this challenge, the present study focuses on the fabrication of flame-retardant and EMI-shielded PLA composites via FDM 3D printing. A series of PLA/IFR/GNPs FR composites with different formulations were designed and prepared. By performing microstructural characterization, thermal analysis, flame retardancy evaluation, and electromagnetic parameter measurements, the synergistic dispersion of fillers, thermal decomposition behavior, flame-retardant mechanisms, and EMI shielding mechanisms were systematically investigated. Furthermore, the effects of FDM printing parameters on the functional performance of the composites were explored. Finally, a synergistic flame retardancy–EMI shielding mechanism model was established, thus providing a reproducible design strategy for the development of high-performance multifunctional 3D-printed materials.

## 2. Materials and Methods

### 2.1. Materials

Polylactic acid (PLA, grade 4032D) was purchased from NatureWorks LCC, Plymouth, MN, USA. Ammonium polyphosphate (APP) and pentaerythritol (PER) were used as ingredients for the intumescent flame-retardant (IFR) system. The IFR was thus composed of APP and PER with a weight ratio of 3:1, where APP served as both the phosphorus source and gas source, while PER acted as the carbon source. Ammonium polyphosphate (APP, degree of polymerization >1500) and pentaerythritol (PER) were supplied by Chengdu Senpoly Biotechnology Co., Ltd., Chengdu, China. Graphene nanoplatelets (GNPs, XFQ022; thickness: 1–4 nm, lateral size: 1–3 μm) were supplied by Nanjing XFNANO Materials Tech Co., Ltd., Nanjing, China.

### 2.2. Preparation of FR PLA-Based Composites

The FR PLA-based composites were prepared by melt compounding. The principle for determining the material formulations is clearly illustrated in the Supporting Information. Dried PLA pellets, the IFR components, and GNPs were first weighed according to the formulations listed in Table 1. The PLA, IFR components, and GNPs were premixed using a high-speed mixer at 1500 rpm for 5 min and then fed into a co-rotating twin-screw extruder (Process 11, Thermo Electron Gembh, Karlsruhe, Germany) through a feeding hopper. The barrel temperatures from the feeding zone to the die were set to 160, 170, 180, 185, 185, and 180 °C, respectively, with a screw speed of 200 rpm. The extrudates were then cooled in a water bath and pelletized into composite pellets with diameters of approximately 2–3 mm. The obtained pellets were subsequently dried in a vacuum oven at 80 °C for 12 h before further processing.

The dried composite pellets were then extruded into FDM filaments with a diameter of 1.75 ± 0.05 mm using a single-screw filament extruder. The temperature profile was set to 170 °C (Zone 1), 180 °C (Zone 2), 185 °C (Zone 3), and 180 °C (Zone 4, die zone). The screw speed was maintained at 15 rpm, while the haul-off speed was automatically adjusted through a closed-loop control system to ensure that the filament diameter remained within the specified tolerance range.

The dried composite filaments were subsequently fabricated into test specimens using a CreatBot F430 NX FDM 3D printer (Henan CreatBot Technology Limited, Zhengzhou, China). The main printing parameters are summarized in Table 2. A nozzle with a diameter of 0.4 mm was used, and the layer height was set to 0.2 mm. The specimens were printed with a raster pattern of −60°/0°/+60° and an infill density of 100%. The bed and nozzle temperatures were maintained at 60 and 210 °C, respectively.

### 2.3. Characterizations

Fourier transform infrared (FTIR) spectra were recorded using an INVENIO R spectrometer (Bruker, Ettlingen, Germany). X-ray photoelectron spectroscopy (XPS) measurements were carried out on a K-Alpha spectrometer equipped with Al Kα radiation. X-ray diffraction (XRD) patterns were obtained using an Ultima IV diffractometer (Rigaku, Akishima, Japan) with Cu Kα radiation (λ = 1.541 Å). The microstructures of the cryo-fractured surfaces, char residues, and deposition interfaces of the printed samples were observed using a field-emission scanning electron microscope (SEM, Apreo S HiVoc, Thermo Fisher Scientific, Hillsboro, OR, USA) operated at an accelerating voltage of 5 kV. An energy-dispersive X-ray spectroscopy system (EDS, Octane Elect Super, EDAX, Mahwah, NJ, USA) was employed for elemental mapping analysis to evaluate the distribution of P and N elements from IFR and C from GNPs. The thermal stability was evaluated using a TGA2 thermogravimetric analyzer (METTLER TOLEDO, Greifensee, Switzerland). Approximately 3–10 mg of sample was placed in a platinum crucible and heated from room temperature to 700 °C at a heating rate of 10 °C min^−1^ under a nitrogen atmosphere (50 mL min^−1^). Each sample was tested in triplicate, and the average values were reported. The flame-retarding properties were comprehensively evaluated by limiting oxygen index (LOI), UL-94 vertical burning, and cone calorimetry tests. The LOI values were measured according to ASTM D2863 [24] using a JF-3 oxygen index tester (Nanjing Jiangning Analytical Instrument Co., Ltd., Nangjing, China). FDM-printed specimens with dimensions of 130 mm × 6.5 mm × 3.0 mm and 100% infill were tested. Five independently prepared specimens were tested for each formulation, and the results are reported as the mean ± standard deviation. The UL-94 vertical burning tests were conducted in accordance with the UL-94 standard [25] using specimens measuring 125 mm × 13 mm × 3.0 mm. Five specimens were tested for each formulation. The cone calorimeter tests were performed using an FTT cone calorimeter under an external heat flux of 35 kW m^−2^. Specimens with dimensions of 100 mm × 100 mm × 3.0 mm were placed horizontally, and the heat release rate (HRR), total heat release (THR), smoke production rate (SPR), and CO/CO_2_ yields were recorded. Three independent specimens were tested for each formulation. The reported quantitative parameters are expressed as the mean ± standard deviation, while a representative curve is shown for clarity. The electromagnetic interference shielding effectiveness (EMI SE) was measured using a vector network analyzer (N5247A, Keysight Technologies, Penang, Malaysia) using the waveguide method in the X-band frequency range (8.2–12.4 GHz). The samples were machined into circular disks with a radius of 12.5 mm and a thickness of 2.0 mm to match the dimensions of the X-band waveguide cavity. Before testing, a full two-port calibration (SOLT) was performed using standard calibration kits. The scattering parameters (*S_11_* and *S_21_*) were measured, and the reflection coefficient (R), transmission coefficient (T), and absorption coefficient (A) were calculated according to the following equations:

(1) $$R={\left|S_{11}\right|}^{2},\;T={\left|S_{21}\right|}^{2},\;A=1-R-T.$$

The total shielding effectiveness (SE_T_), reflection shielding effectiveness (SE_R_), and absorption shielding effectiveness (SE_A_) were calculated using the following equations:

(2) $${SE}_{T}=-10lg\left(T\right),\;{SE}_{R}=-10lg\left(1-R\right),\;{SE}_{A}=-10lg\left(\frac{T}{1-R}\right).$$

To intuitively evaluate the shielding mechanism, the contribution ratio of shielding effectiveness is commonly used:

(3) $$SE_{R}\;(\%)\;=\;\frac{SE_{R}}{SE_{T}}\;\times \;100\%;\;SE_{A}\;(\%)\;=\;\frac{SE_{A}}{SE_{T}}\;\times \;100\%.$$

Three independently printed specimens were tested for each formulation, with measurements performed at five different positions on each specimen. The average shielding effectiveness was reported. The average shielding effectiveness curve was then obtained. The complex permittivity (ε′ and ε″) spectra of selected samples were calculated from the measured S-parameters using a waveguide method in the X-band frequency range (8.2–12.4 GHz). Samples with dimensions of approximately 22.9 mm × 10.2 mm × 2.0 mm were prepared to fit the X-band waveguide fixture.

The electrical conductivity of FR PLA-based composites was measured using a low-resistance dual-electrode tester and a high-resistance four-point probe conductivity tester (FT-361A and FT-371Ar, Ningbo Ruikewei Intelligent Technology Co., Ltd., Ningbo, China). The appropriate testing mode was selected according to the conductivity range of each sample. For each formulation, one specimen was measured at five different positions, and the average value was reported. The interlayer shear strength of the printed specimens was measured using a universal testing machine (Instron 5967, Instron, Norwood, MA, USA) based on a short-beam shear test method. The specimens had dimensions of 40 mm × 10 mm × 4 mm. The loading direction was perpendicular to the printing direction, and the crosshead speed was set to 1.0 mm min^−1^. At least five specimens were tested for each formulation, and the average value together with the standard deviation was reported.

## 3. Results

### 3.1. FDM 3D Printing Processability of FR PLA-Based Composites

The printability of the FR PLA-based composites was assessed using filaments with different GNPs loadings. The extrusion stability, filament diameter variation, surface morphology, and printing behavior were used as the main evaluation criteria. The evaluation results are summarized in Table 3. As can be seen, when the GNPs content was ≤3 wt%, the filaments could be printed normally, but when the GNPs content reached 5 wt%, the printing became relatively difficult.

The interlayer shear strength results are summarized in Table 4. Within the PLA/IFR15 sample, the moderate GNPs incorporation improved the interlayer shear strength of the printed specimens, indicating enhanced bonding between adjacent deposited layers [26]. Under the conditions of this study, 3.0 wt% was identified as the optimal GNPs loading, which resulted in approximately a 10.5% increase in interlayer shear strength compared with the control sample. When the GNPs content exceeded 3.0 wt%, the reinforcing effect gradually diminished owing to filler agglomeration; nevertheless, the interlayer shear strength was higher than that of the specimen without GNPs [27].

**Figure 1:**
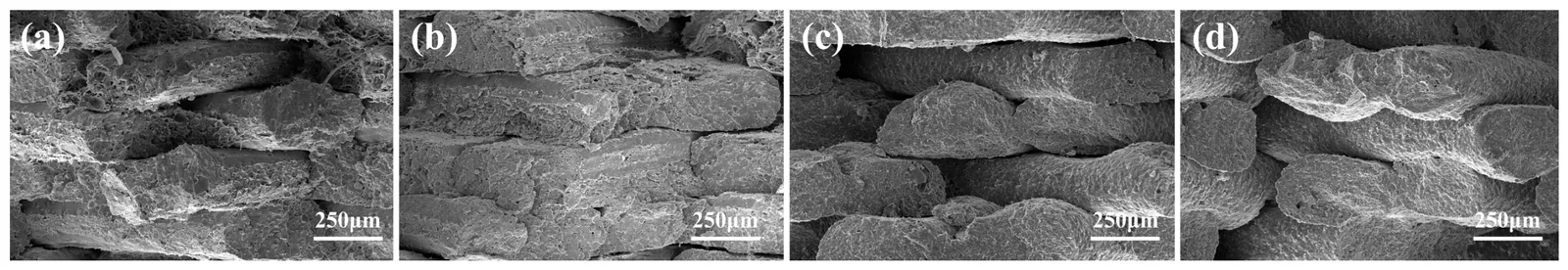
SEM images of deposition interfaces of printed specimens: (**a**) PLA/IFR15; (**b**) PLA/IFR15/GNPs1; (**c**) PLA/IFR15/GNPs3; (**d**) PLA/IFR15/GNPs5.

The cryo-fractured cross-sectional morphologies of 3D-printed filaments with varying GNPs contents were examined by scanning electron microscopy (SEM), and the results are presented in Figure 1. For the PLA/IFR15 control sample without GNPs (Figure 1a), distinct interfacial gaps and numerous micropores were observed between adjacent deposited filaments. Formation of these structural defects indicates insufficient melt interdiffusion and weak interlayer adhesion during FDM printing [28,29]. After the addition of 1.0 wt% of GNPs (Figure 1b), the interfacial gaps became notably narrower and the micropores decreased, suggesting an initial improvement in interlayer fusion and bonding. When the GNPs content increased to 3.0 wt% (Figure 1c), the interlayer interface exhibited the most favorable morphology, with tightly fused deposited filaments, indistinct layer boundaries, and minimized pore defects. However, upon further increasing the GNPs content to 5.0 wt% (Figure 1d), the number of interfacial gaps and pores increased again. This deterioration can be attributed to the agglomeration of excessive GNPs, which hinders melt interdiffusion and spreading between adjacent layers while simultaneously creating stress concentration sites, thereby reducing interlayer bonding strength [26,30].

### 3.2. Structural Characterization of FR PLA-Based Composites

**Figure 2:**
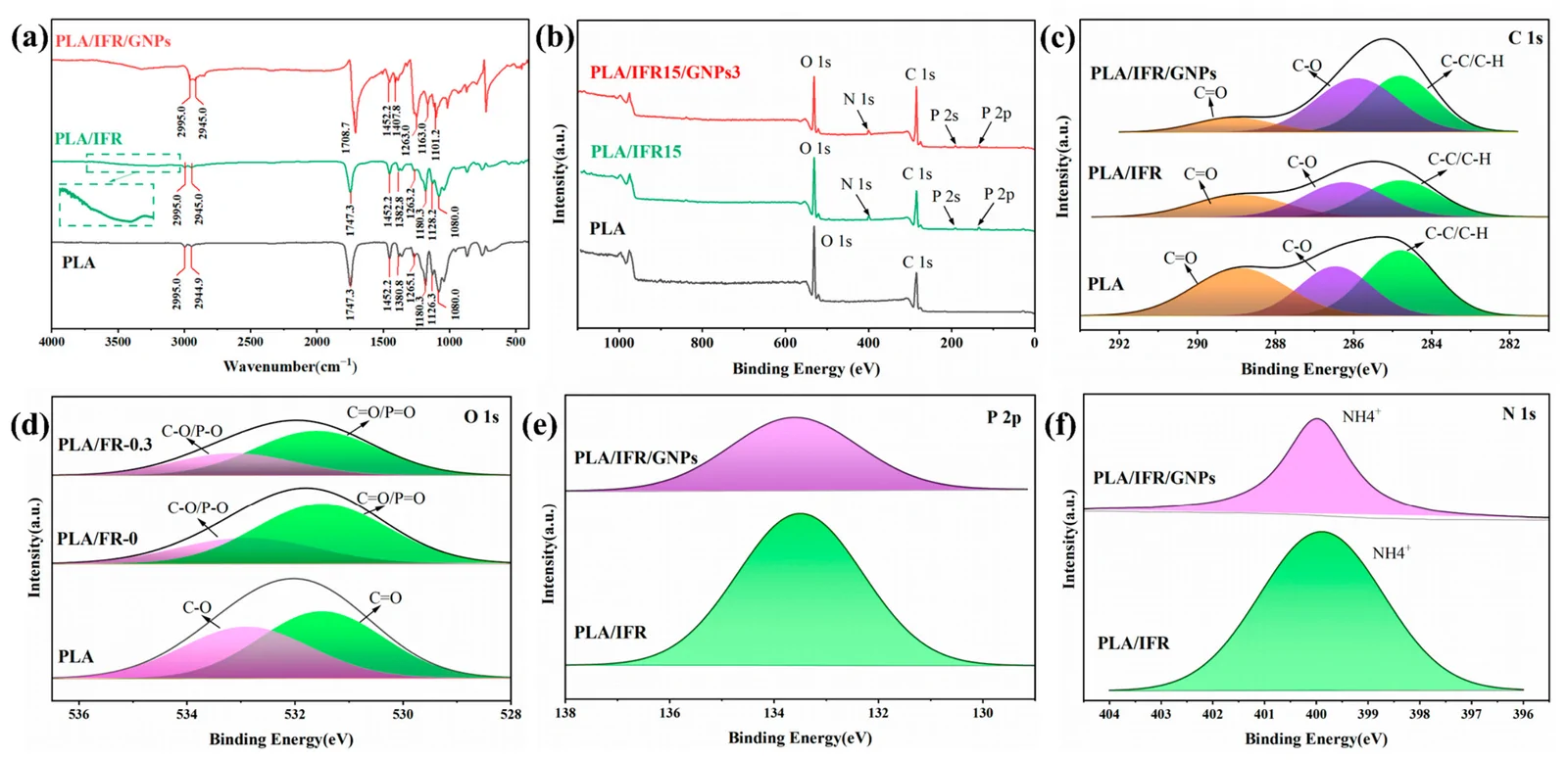
(**a**) FTIR full spectra; (**b**) XPS survey spectra; high-resolution XPS spectra of (**c**) C 1s; (**d**) O 1s; (**e**) P 2p and (**f**) N 1s for PLA, PLA/IFR, and PLA/IFR/GNPs samples.

To elucidate the chemical structure of the composites, FTIR and XPS analyses were performed, as shown in Figure 2. For the PLA matrix, characteristic absorption peaks appeared at 1747.5 cm^−1^ (C=O stretching), 1180.4, 1127.1, and 1080.2 cm^−1^ (C–O–C stretching), and at 2994.9 and 2946.4 cm^−1^ (C–H stretching). After the incorporation of IFR (composed of APP and PER), a broad absorption band emerged at 3200–3400 cm^−1^ (O–H and N–H stretching). Additionally, new peaks were observed at 1266.1 cm^−1^ (P=O stretching) and 1015 cm^−1^ (C–O stretching of PER primary alcohol), confirming the successful introduction of the acid and carbon sources [31,32]. Upon the addition of GNPs, no new absorption peaks appeared; however, the C=O stretching peak of PLA exhibited a red shift, indicating the formation of hydrogen bonding interactions between the ester carbonyl groups of PLA and the surface functional groups of APP, PER, and GNPs [33]. Overall, the FTIR results confirmed the presence of intermolecular interactions between the components.

Figure 2b presents the XPS survey spectra of PLA, PLA/IFR15, and PLA/IFR15/GNPs3 composites. For neat PLA, only the characteristic C 1s and O 1s peaks were detected, corresponding to the carbon and oxygen atoms in the PLA molecular chains. After the incorporation of IFR, distinct N 1s and P 2p peaks appeared in the PLA/IFR15 sample, indicating the successful introduction of APP into the PLA matrix. Compared with PLA/IFR15, the PLA/IFR15/GNPs3 sample retained prominent N and P signals, while the intensity of the C 1s peak increased, which can be mainly attributed to the presence of GNPs. For the C 1s spectra, three characteristic components were identified at approximately 284.7, 286.0–286.4, and 288.8–289.0 eV, corresponding to C–C/C–H, C–O, and C=O groups, respectively. Compared with neat PLA, the incorporation of IFR altered the relative contributions of the C–O and C=O components, suggesting interfacial interactions between PLA and the APP/PER flame-retardant system [34,35]. After further addition of GNPs, the relative contribution of the C–C/C–H component increased due to the sp^2^-hybridized carbon structure of GNPs. Moreover, slight shifts in the oxygen-containing carbon peaks indicate a redistribution of electron density among PLA, IFR, and GNPs. For the O 1s spectra, neat PLA could be deconvoluted into two components centered at approximately 531.5 and 532.9 eV, assigned to the carbonyl oxygen (C=O) and ether oxygen (C–O) in the ester groups, respectively. Upon introduction of IFR, contributions from the P=O and P–O moieties in APP became discernible. Accordingly, the O 1s envelope could be deconvoluted into two major components, corresponding to C=O/P=O and C–O/P–O–C (P–O–P) oxygen environments [36]. Compared with neat PLA, the PLA/IFR sample exhibited an increased proportion of the lower-binding-energy component, confirming the successful incorporation of phosphorus-oxygen species. Subsequent addition of GNPs induced slight shifts in the O 1s peak positions, further evidencing interfacial interactions and electron density redistribution among PLA, IFR, and GNPs. In the P 2p spectra, both PLA/IFR15 and PLA/IFR15/GNPs3 exhibited a characteristic peak at approximately 135 eV, which is assigned to the P–O/P=O species derived from APP. This result confirms the presence of phosphorus-containing species in the PLA matrix. Similarly, the N 1s spectra of both composites showed a single peak centered at approximately 401 eV, corresponding to the NH_4_^+^ species in APP. These results further verify the successful incorporation of the APP-based intumescent flame-retardant system.

**Figure 3:**
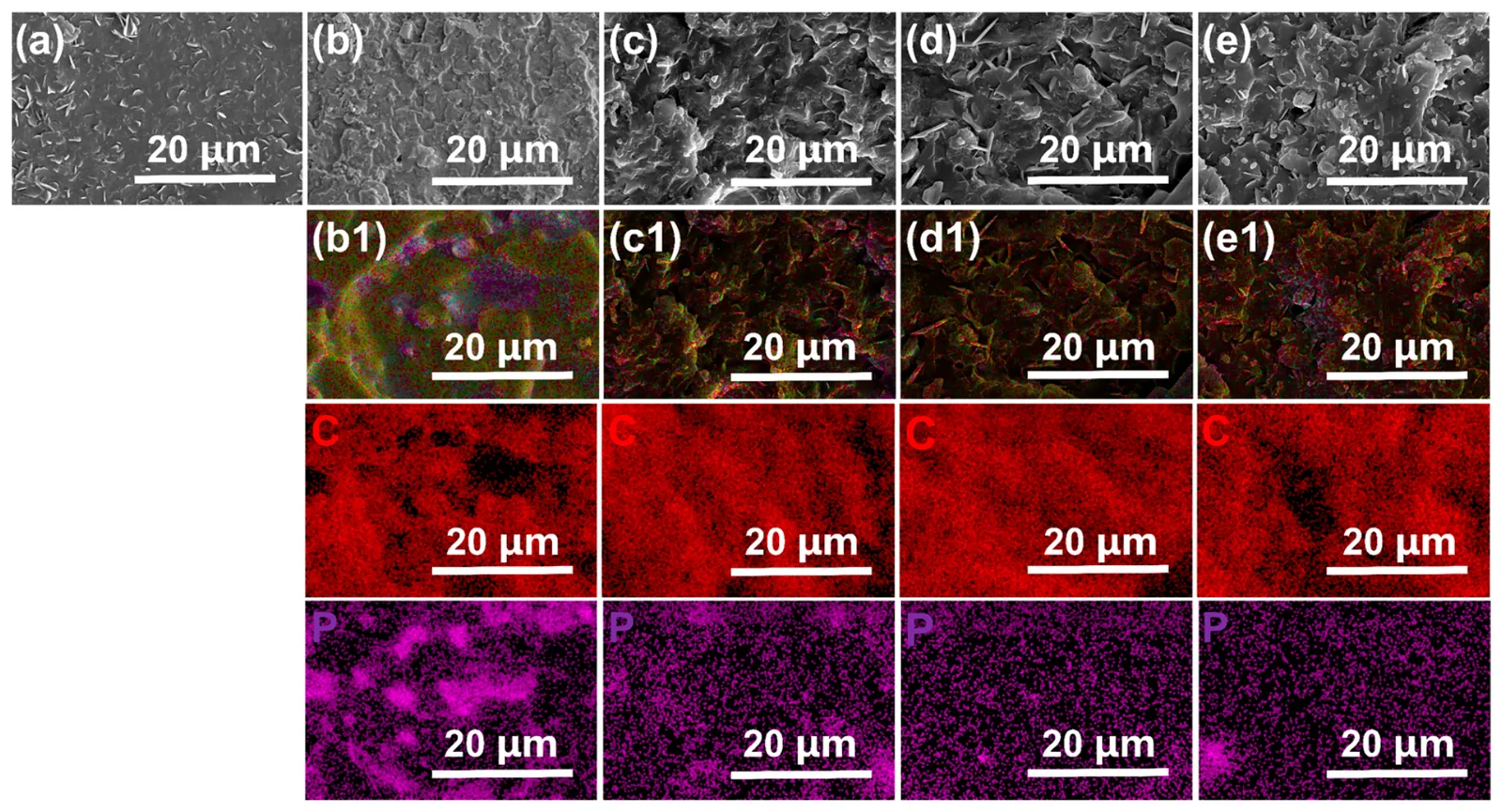
SEM morphologies of fracture surfaces and EDS element distribution of different FR PLA-based composites: (**a**) PLA/GNPs; (**b**) PLA/IFR; (**c**–**e**) PLA/IFR/GNPs1, PLA/IFR/GNPs3, and PLA/IFR/GNPs5, respectively; (**b1**–**e1**) corresponding to the EDS element distribution of PLA/IFR, PLA/IFR/GNPs1, PLA/IFR/GNPs3, and PLA/IFR/GNPs5 samples.

To further verify the synergistic dispersion behavior between the flame-retardant and conductive filler, the relative contents of the IFR and GNPs were varied. The cryo-fractured cross-sections of the composites were characterized by SEM and EDS to analyze the microstructural morphology and elemental distribution, thereby evaluating the internal structural homogeneity of the composites. The results are shown in Figure 3. As presented in Figure 3b,e, the addition of GNPs contributes to improved dispersion of the IFR. In the EDS elemental maps, larger P-element agglomerates are observed in PLA/IFR15, indicating relatively poorer IFR dispersion in the absence of GNPs [37]. This suggests that GNPs not only disperse well themselves but also facilitate the dispersion of IFR. This synergistic dispersion effect can be attributed to two factors: (i) steric hindrance, whereby GNPs physically inhibit contact between IFR particles and reduce their agglomeration tendency [38], and (ii) interfacial affinity, where π–π stacking interactions between the graphitic structure of GNPs and the aromatic or polar groups in IFR, along with possible hydrogen bonding, may promote the formation of a more homogeneous filler distribution [39].

Furthermore, as shown in Figure 3c, at a low GNPs loading (1 wt%), the GNPs are insufficient to fully cover the IFR particles, leaving portions of their surfaces exposed; nevertheless, the dispersion is still improved compared with the GNPs-free system. At a moderate GNPs content (3 wt%), the IFR particles are uniformly coated by GNPs, forming a relatively homogeneous microstructure. When the GNPs content is further increased to 5 wt%, the excess GNPs tend to aggregate into micron-scale clusters, leading to locally enhanced conductivity but an inhomogeneous distribution of the flame-retardant components.

### 3.3. Thermogravimetric Analysis

**Figure 4:**
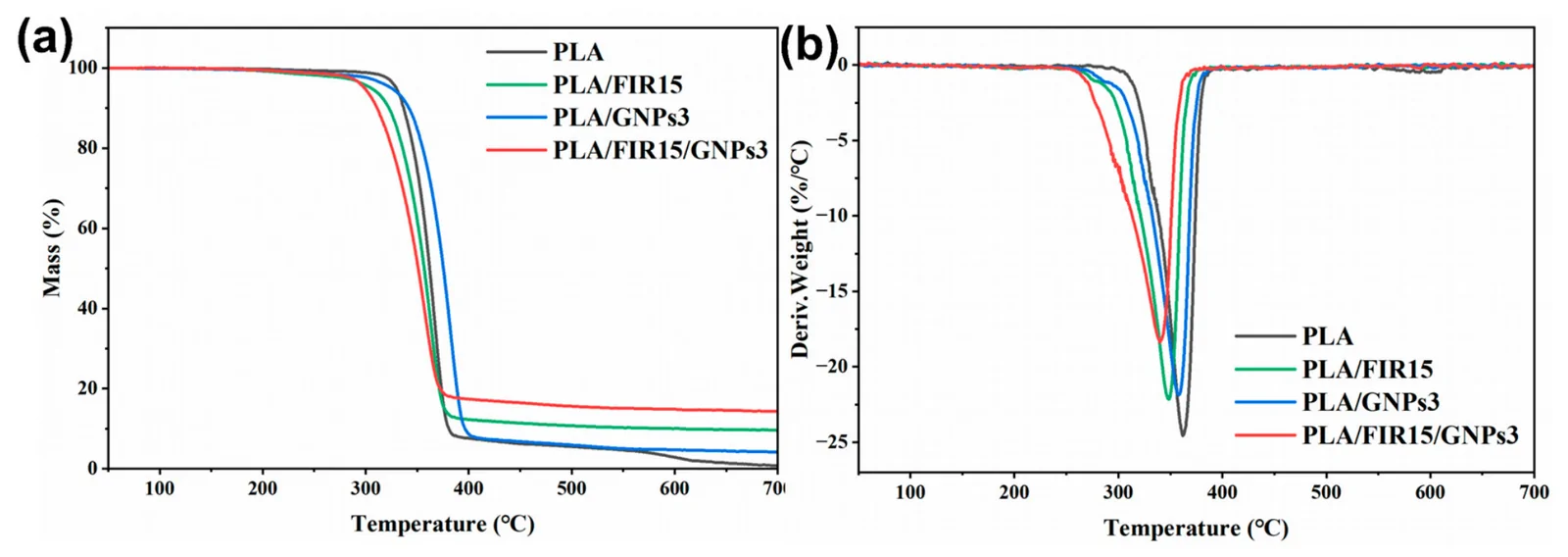
(**a**) TG and (**b**) DTG curves of PLA, PLA/IFR15, PLA/GNPs3, and PLA/IFR15/GNPs3 samples.

The thermal degradation behaviors of the composites were further examined by thermogravimetric analysis (TGA). Figure 4 shows the TGA and derivative thermogravimetry (DTG) curves of PLA, PLA/IFR15, PLA/GNPs3, and PLA/IFR15/GNPs3 under a nitrogen atmosphere, and the corresponding characteristic parameters are summarized in Table 5. For neat PLA, the initial decomposition temperature (T_5%_) and the maximum degradation temperature (T_max_) are 329.5 °C and 361.8 °C, respectively. After addition of IFR, the T_5%_ and T_max_ values decrease to 305.2 °C and 348.5 °C, respectively. For the dual-filler (IFR/GNPs) system, the T_5%_ and T_max_ values decrease to 298.7 °C and 340.2 °C, respectively. Clearly, the samples containing IFR, including PLA/IFR15 and PLA/IFR15/GNPs3, have a lower thermal stability than neat PLA, and this exactly reflects the flame-retarding effect of IFR through its timely degradation from a very early stage (forming protective carbonaceous layers). This is a common phenomenon, and similar changes have been well-reported in the literature [40]. The PLA/GNPs3 sample only experiences lower thermal stability relative to neat PLA in a very short period at the initial stage, and soon exceeds that of neat PLA, possibly due to its promotional effect on PLA degradation. The reason for the above changes is that APP begins to thermally decompose at a relatively low temperature and generates ammonia gas, water vapor, and polyphosphoric acid species [35]. These acidic species then promote the dehydration and degradation of PLA and the carbonization reaction with PER, thus initiating char formation at an earlier stage. The incorporation of GNPs alone (PLA/GNPs3) causes only a slight decrease in T_5%_ and T_max_, whilst on the contrary, the ternary PLA/IFR15/GNPs3 system shows a much more significant decrease because the early catalytic char-forming process of the IFR dominates at the initial degradation stage.

Regarding the char yield, neat PLA almost completely decomposed, leaving a residual char yield of less than 1%. The char yield of PLA/IFR15 increased to 9.6%, attributed to the formation of an expanded char layer during thermal decomposition of IFR. PLA/GNPs3 exhibited a char yield of 4.1% owing to the excellent thermal stability of GNPs. The dual-filler system exhibited the highest char yield of 14.3%. This result suggests that the presence of GNPs favors the stabilization of the IFR-derived carbonaceous structure during thermal degradation [41].

### 3.4. Flame-Retardant Properties and Mechanism

**Figure 5:**
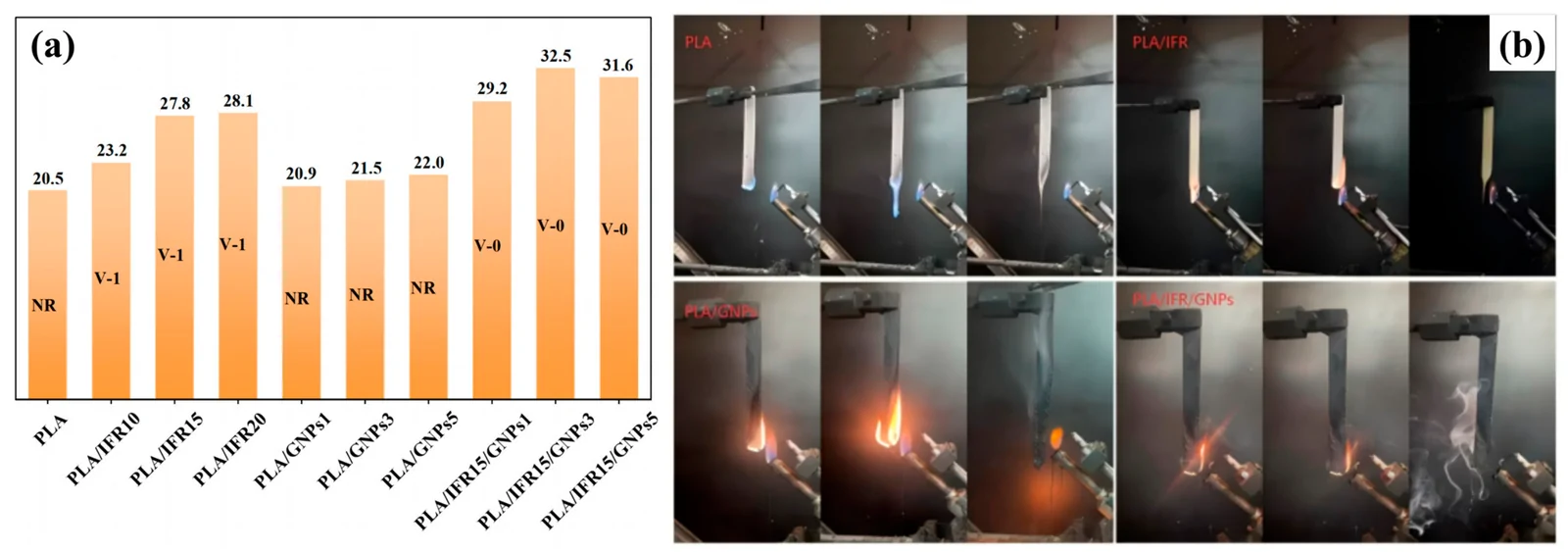
(**a**) LOI and UL-94 test results of different FR PLA-based composites; (**b**) digital photographs.

The flame-retardant properties of the FR PLA-based composites were evaluated by limiting oxygen index (LOI) and UL-94 vertical burning tests, and the results are presented in Table 6 and Figure 5. Neat PLA exhibited an LOI of only 20.5%, indicating its highly flammable nature. With the addition of IFR alone, the LOI increased progressively with IFR content, reaching 27.8% at 15 wt% IFR; a further increase to 20 wt% yielded only a marginal improvement (28.1%), suggesting that the flame-retardant efficiency of IFR approaches saturation at around 15 wt%. In contrast, the incorporation of GNPs alone had a limited effect on the LOI, with only a slight rise to 22.0% at a GNPs content of 5 wt%, indicating that GNPs alone offer relatively weak flame-retardant capability. However, when IFR and GNPs were combined, the LOI of PLA/IFR15/GNPs3 reached 32.5%, corresponding to a self-extinguishing level (LOI > 27%). This LOI value is substantially higher than that of the composite containing either IFR or GNPs alone. Thus, the above result suggests a positive synergistic effect between IFR and GNPs rather than a simple additive effect [42]. When the GNPs content was further increased to 5 wt%, the LOI slightly decreased to 31.6%, which may be ascribed to GNPs agglomeration that impaired the uniform char-forming process of IFR.

In the UL-94 vertical burning test, neat PLA sustained combustion after ignition, accompanied by severe dripping that ignited the cotton, resulting in a no-rating (NR) classification. Upon the addition of 15 wt% IFR, the sample self-extinguished with a total afterflame time of 12 s. However, dripping was still observed, and the sample achieved a V-1 rating (single afterflame <30 s, total afterflame <250 s, and no cotton ignition). For the sample containing only 3 wt% GNPs, persistent combustion was observed, and the material remained unrated (NR), further confirming that GNPs alone impart limited flame-retardant performance. In contrast, when IFR and GNPs were combined, all composite samples achieved a V-0 rating (single afterflame <10 s and total afterflame <50 s). Notably, PLA/IFR15/GNPs3 exhibited a total afterflame time of only 3 s, indicating excellent self-extinguishing behavior. As the GNPs content increased from 1 wt% to 3 wt%, the afterflame time decreased, whereas a slight increase was observed at 5 wt%, although the V-0 rating was maintained in all cases.

To quantitatively evaluate the flame-retardant efficiency of the fillers, a flame-retardant efficiency factor (FEF) was defined as follows [43]:

(4) $$FEF=\frac{LOI_{composite}-LOI_{PLA}}{Total\;filler\;content\;(wt\%)}$$

The calculated FEF values are as follows: 0.49 for PLA/IFR15, 0.33 for PLA/GNPs3, and 0.67 for PLA/IFR15/GNPs3. The FEF of the ternary system (0.67) is markedly higher than those of the single-filler systems (0.49 for IFR alone and 0.33 for GNPs alone), clearly indicating that the ternary formulation exhibits a higher flame-retardant efficiency than corresponding single-filler systems. To further quantitatively evaluate whether the combined flame-retardant effect of IFR and GNPs exceeds the sum of their individual contribution, a synergistic efficiency factor (SEF) was adopted based on the synergistic effectivity approach reported by Holdsworth et al. [44]. The SEF was calculated as follows:

(5) $$SEF=\frac{{LOI}_{IFR+GNPs}-{LOI}_{PLA}}{{(LOI}_{IFR}{-LOI}_{PLA})+({LOI}_{GNPs}{-LOI}_{PLA})}$$

where LOI_IFR+GNPs_, LOI_IFR_, LOI_GNPs_, and LOI_PLA_ represent the LOI values of PLA/IFR15/GNPs3, PLA/IFR15, PLA/GNPs3, and neat PLA, respectively. An SEF value greater than 1 indicates a synergistic effect, an SEF value equal to 1 indicates an additive effect, and an SEF value below 1 indicates an antagonistic effect. By calculation, the obtained SEF value for PLA/IFR15/GNPs3 is 1.45, demonstrating that the experimentally observed LOI enhancement of the PLA/IFR15/GNPs3 system exceeds the sum of the individual contributions of IFR and GNPs, confirming a positive synergistic flame-retardant effect between IFR and GNPs.

**Figure 6:**
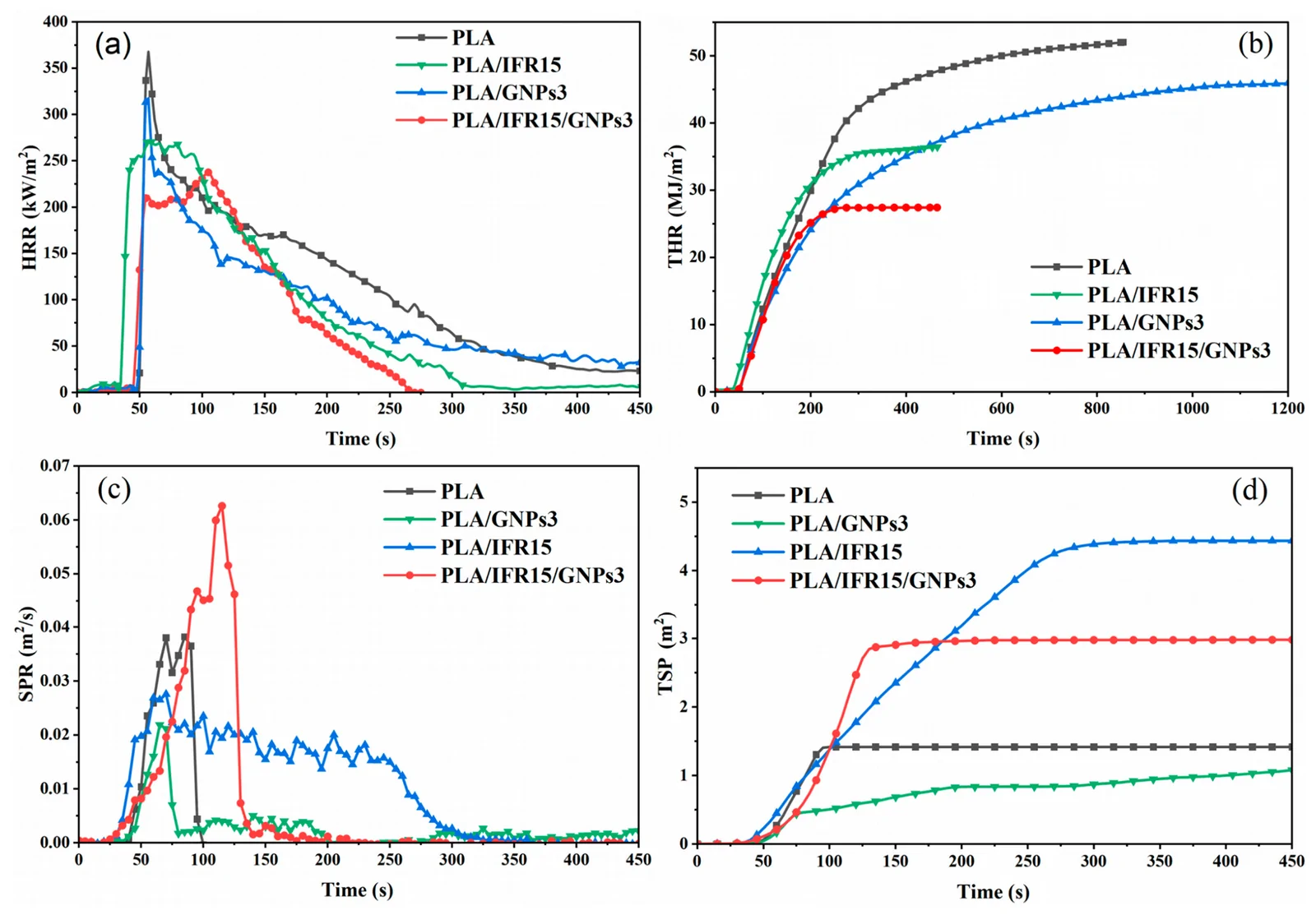
Combustion performance curves of different materials: (**a**) HRR curves; (**b**) THR curves; (**c**) SPR curves; and (**d**) TSP curves.

Cone calorimetry was further employed to evaluate the combustion behavior of the PLA/IFR/GNPs systems, and the heat and smoke release data are presented in Figure 6 and Table 7. Figure 6a,b shows the heat release rate (HRR) and total heat release (THR) curves for neat PLA, PLA/IFR15, PLA/GNPs3, and PLA/IFR15/GNPs3. Neat PLA exhibited a sharp HRR peak (pHRR) of 336.8 kW/m^2^ shortly after ignition, followed by a rapid decline, indicating intense combustion and strong heat feedback. Upon the addition of 15 wt% IFR, the pHRR decreased by approximately 19.7% to 270.4 kW/m^2^, and the HRR curve became broader, suggesting that the expanded char layer formed by IFR can retard the release of pyrolysis volatiles to some extent. For the sample with only 3 wt% GNPs, the pHRR was 313.3 kW/m^2^, corresponding to a reduction of only 7.0% over neat PLA. The HRR curve profile was similar to that of neat PLA, suggesting that GNPs alone were insufficient to form an effective protective char layer. In contrast, the PLA/IFR15/GNPs3 system exhibited a pHRR of 237.6 kW/m^2^, 29.4% lower than that of neat PLA. Notably, two distinct plateaus were observed in the HRR curve: the first small peak corresponds to the early decomposition and char formation of the IFR, whereas the second broader peak is associated with the slow oxidation of the residual char layer. The THR decreased from 52.0 MJ/m^2^ for neat PLA to 27.4 MJ/m^2^ for the synergistic system, representing a reduction of approximately 47.3%. These results demonstrate that the synergistic system effectively suppresses the total heat release during combustion.

The smoke-release behaviors are shown in Figure 6c,d. As can be seen, compared with neat PLA, the PLA/IFR15 composite exhibited an increased pSPR from 0.038 to 0.058 m^2^/s and an increased TSP from 1.4 to 4.4 m^2^. This increase in smoke release can be attributed to the formation of intumescent char layers and oxygen dilution effects (NH_3_ and other non-flammable gases released from APP decomposition dilute the oxygen concentration in the flame zone). They can both suppress the complete oxidation of volatile degradation products during combustion, thus favoring the formation of soot particles rather than full conversion to CO_2_ and H_2_O. In contrast, the addition of GNPs alone reduced both the pSPR and TSP to 0.022 m^2^/s and 1.1 m^2^, respectively, indicating a smoke-suppressing effect of GNPs in the PLA matrix. When IFR and GNPs were combined, the PLA/IFR/GNPs system exhibited a further increase in residual char yield to 25.7%. Meanwhile, the TSP was markedly reduced to 3.0 m^2^ (31.8% lower than that of PLA/IFR), while the pSPR showed only a slight increase to 0.063 m^2^/s, indicating that GNPs reduced the cumulative smoke release of the IFR-containing system but did not suppress the maximum instantaneous smoke release. This behavior may be related to the formation of a more compact and stable protective char layer promoted by the co-existence of GNPs and IFR [45], which strengthens the condensed-phase barrier and partially reduces the release of smoke-producing degradation products.

**Figure 7:**
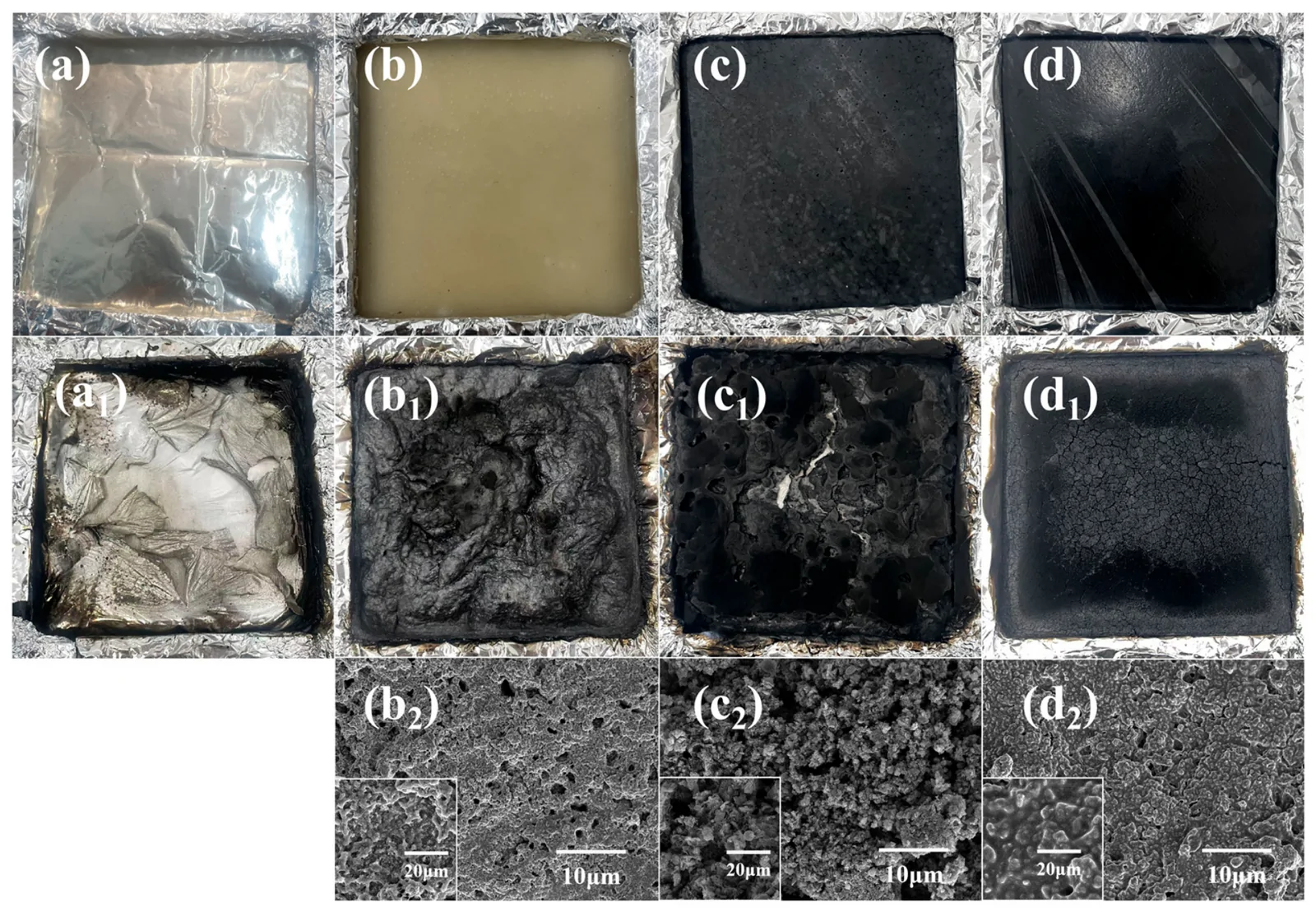
(**a**–**d**,**a1**–**d1**) Digital photos and (**b2**–**d2**) SEM images of the residues of (**a**) PLA; (**b**) PLA/IFR; (**c**) PLA/GNPs; and (**d**) PLA/IFR/GNPs samples.

The aforementioned flame-retardant results indicate that the improved flame retardancy of the PLA/IFR/GNPs system is closely associated with the reinforced condensed-phase char layer. The residual chars obtained after cone calorimetry were further examined to correlate their morphology with the observed combustion behavior. Figure 7 presents digital photographs and SEM images of the residual chars. Neat PLA produced almost no char residue, leaving only a small amount of ash on the aluminum foil. PLA/IFR15 formed an expanded char layer with a thickness of approximately 3–5 mm and a rough, uneven surface, while SEM images revealed micron-scale porous structures within the char. For PLA/GNPs3, only a small amount of char residue was observed; the char layer was thin and discontinuous, insufficient to provide an effective barrier, and SEM images showed a porous, open structure. In contrast, the PLA/IFR15/GNPs3 system formed a more compact expanded char layer with significantly fewer cracks. SEM images revealed a continuous and uniform char structure, indicating enhanced mechanical integrity and improved thermal-oxidative stability of the char layer.

**Figure 8:**
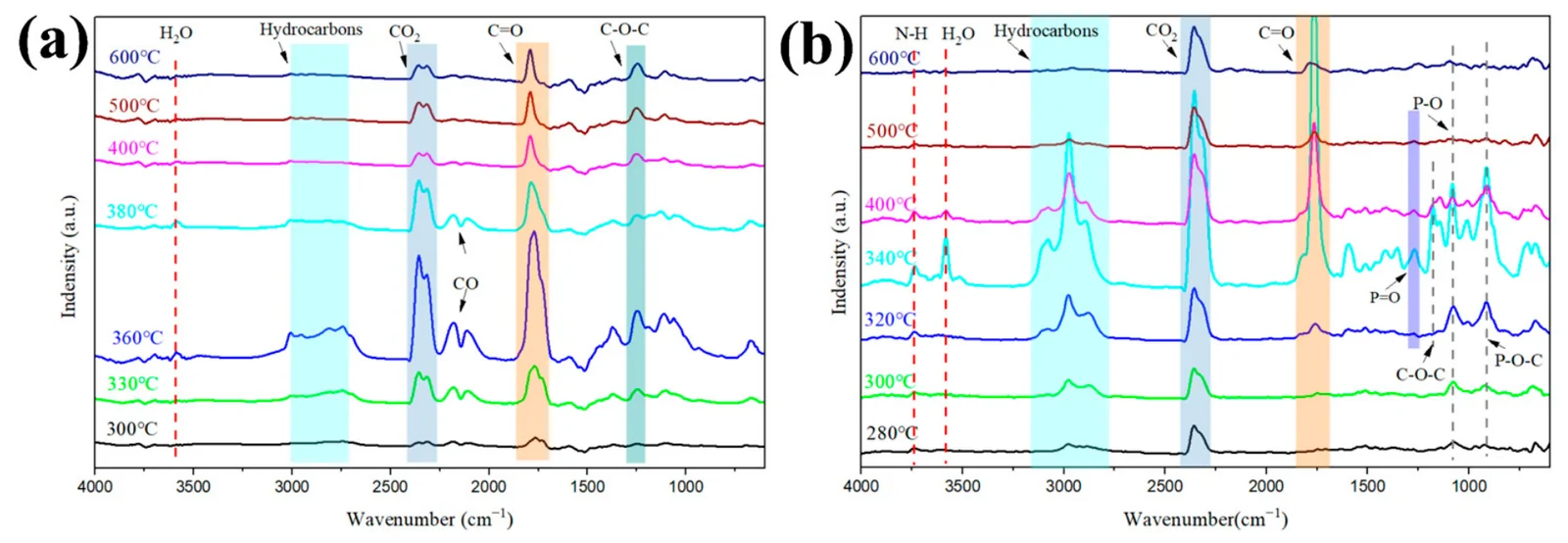
TG-IR spectra of the pyrolysis products of (**a**) PLA and (**b**) PLA/IFR/GNPs at different temperatures.

To further elucidate the flame-retardant mechanism and thermal degradation process of PLA/IFR15/GNPs3, the pyrolysis products generated at different temperatures were analyzed, and the results are presented in Figure 8.

In the TG-IR results (Figure 8), almost no decomposition products of PLA were detected below 300 °C. As the temperature increased, the PLA began to decompose at around 330 °C and reached its maximum decomposition rate at 380 °C. The main decomposition products of PLA include hydrocarbons (2963–2925 cm^−1^ and 1456–1381 cm^−1^), carbonyl-containing compounds (1626 cm^−1^), CO (2106–2200 cm^−1^), CO_2_ (2360 cm^−1^), and C–O-containing species (1180–1260 cm^−1^). For the PLA/IFR/GNPs system, in addition to the characteristic signals of PLA pyrolysis products, several new absorption peaks were observed. At approximately 280 °C, a characteristic N–H absorption band appeared (3676–3747 cm^−1^), along with phosphorus-containing pyrolysis products such as P=O (1281 cm^−1^), P–O (1076 cm^−1^), and P–O–P (914 cm^−1^). The intensity of these signals increased significantly at elevated temperatures (above 320 °C) [46]. These phosphorus-containing fragments originate from the decomposition of IFR. Combined with the TGA results, it is indicated that these phosphorus-containing species are generated before PLA decomposition and participate in the early char-forming process. This effect contributes to reducing the subsequent combustion intensity of PLA, which is in good agreement with the cone calorimetry results.

The thermal, gaseous-product, and char-residue analyses jointly suggest that the flame-retarding process evolves through three successive stages. In the range of 280–300 °C, APP begins to decompose, producing NH_3_, vapor, and phosphorus-containing acidic species, while the PLA matrix is also in its initial degradation stage. The generated H_3_PO_4_ catalyzes the dehydration of PLA, forming unsaturated char precursors [47]. Meanwhile, polar functional groups on the GNPs surface promote the adsorption of phosphoric acid species, thereby accelerating the char-forming process. As the temperature rises to 300–400 °C, the resulting gaseous products from APP promote the expansion of the formed carbonaceous layer. GNPs become anchored on the bubble walls of the expanded char, suppressing bubble coalescence and rupture and thus yielding a more stable char structure. Above 400 °C, the expanded residue develops into a more continuous barrier layer that limits direct heat and mass exchange between the underlying material and the combustion environment. Meanwhile, the GNPs network acts as a thermal conduction pathway, dissipating localized heat and preventing the formation of hot spots that could otherwise cause char layer failure.

The flame-retardant behavior of the system can be attributed to the combined contributions of the gas and condensed phases. Gas-phase flame retardancy is primarily achieved through radical quenching (•OH and •H) and dilution of combustible gases. Upon heating, APP releases non-combustible gases such as NH_3_ and water vapor, which dilute oxygen and combustible volatiles and disturb radical chain reactions in the flame zone. However, in systems with relatively high LOI values (>30%), the gas-phase contribution is generally not the dominant factor.

The condensed-phase mechanism is mainly governed by the formation of a protective char layer that hinders heat and mass transfer. In this process, APP acts as an acid source that catalyzes PLA dehydration and char formation, while PER serves as a carbon source, together forming a phosphorus-rich intumescent char layer. GNPs play a multifunctional role: (i) they enhance the mechanical integrity of the char layer by acting as reinforcing fillers that improve crack resistance under prolonged heat flux; (ii) provide a physical barrier effect by increasing the tortuosity of gas diffusion pathways within the char layer, thereby delaying the release of flammable volatiles; and (iii) improve thermal stability, as GNPs possess high thermal-oxidative stability (maintaining structural integrity up to 600 °C in air) and serve as a structural skeleton that prevents collapse of the char layer.

### 3.5. Electromagnetic Interference Shielding Performance and Mechanism

**Figure 9:**
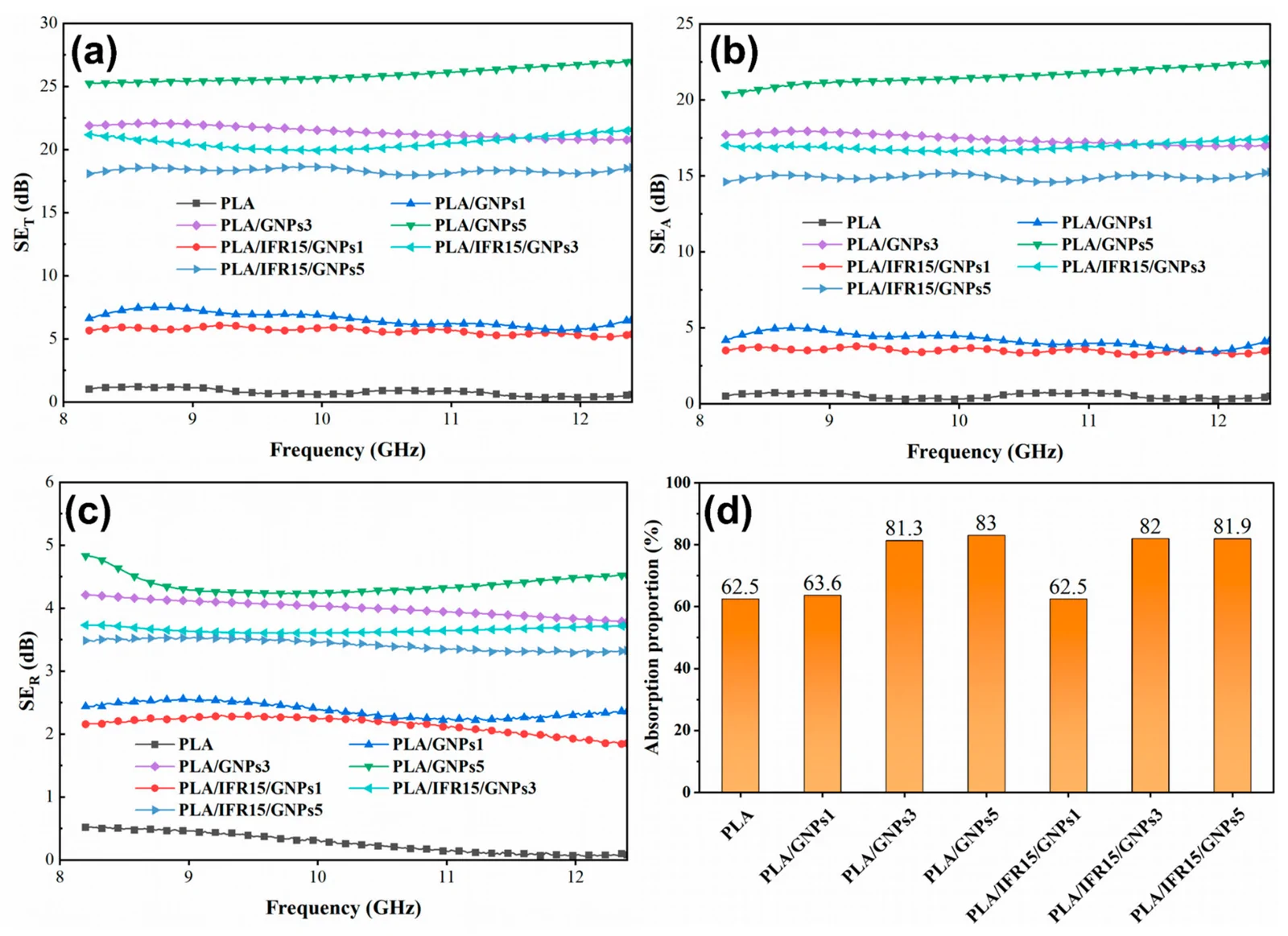
EMI shielding performance of different samples in the X-band. (**a**) SE_T_; (**b**) SE_A_; and (**c**) SE_R_ values of different samples. (**d**) Absorption contribution ratio of different samples.

The EMI shielding behaviors of the composites were evaluated over the X-band frequency range of 8.2–12.4 GHz. Figure 9 presents the frequency-dependent total shielding effectiveness (SE_T_), absorption shielding effectiveness (SE_A_), and reflection shielding effectiveness (SE_R_) curves for PLA, PLA/GNPs3, PLA/IFR15, and PLA/IFR15/GNPs3. Table 8 summarizes the SE_T_, SE_A_, and SE_R_ values over the X-band.

The SE_T_ of neat PLA remained close to 0 dB throughout the X-band, consistent with its electrically insulating character. With the addition of GNPs alone, the SE_T_ increased from 6.6 dB to 25.9 dB as the GNPs content was raised from 1 wt% to 5 wt%. This enhancement was attributed to the gradual formation of a conductive network, which promoted electromagnetic wave attenuation through reflection and absorption [48]. At a GNPs content of 3 wt%, the SE_T_ reached 21.4 dB, meeting the commercial requirement for EMI shielding applications (>15 dB) and confirming that the increased conductive filler effectively attenuated electromagnetic waves. Meanwhile, the contribution of absorption shielding effectiveness (SE_A_) to the total shielding effectiveness gradually increased, with the absorption ratio rising from 63.6% to 83.0%. This trend indicates an increasingly pronounced absorption-dominated shielding behavior with increasing GNPs content. Upon the incorporation of IFR, the overall shielding performance of the PLA/IFR/GNPs system decreased slightly (e.g., for the GNPs5 system, the SE_T_ dropped from 25.9 dB to 18.3 dB), which may be ascribed to the insulating nature of IFR disrupting the conductive network. However, the absorption contribution increased (up to 82.0%), suggesting that the incorporation of IFR introduces additional heterogeneous interfaces into the conductive network, which may enhance interfacial polarization and provide additional internal scattering pathways for electromagnetic waves. These effects may contribute to the relatively high absorption contribution observed for the PLA/IFR/GNPs composites. On the other hand, it should be noted that although the FDM-constructed layer interfaces and interlayer pores could provide additional scattering sites for electromagnetic waves, the quantitative contribution of multiple internal reflections (SE_M_) could be negligible in the present material, as the SE_T_ value (20.6 dB) exceeds the commonly accepted threshold of 15 dB, above which the SE_M_ can be reasonably ignored [49]. Overall, GNPs primarily contribute to conduction loss and overall shielding effectiveness, whereas IFR optimizes the absorption mechanism, rendering the composite more absorption-dominated in its EMI shielding behavior. It is worth noting that the SE_T_ of PLA/IFR15/GNPs5 is lower than that of PLA/IFR15/GNPs3. Combined with the earlier SEM analysis of cryo-fractured surfaces, this observation can be attributed to two factors: (i) aggregation of excess GNPs, and (ii) insulating IFR particles hindering effective contact between GNPs, leading to discontinuous conductive pathways [12,49,50,51]. On the whole, PLA/IFR15/GNPs3 exhibits the best overall performance, with an SE_T_ of 20.6 dB, an absorption contribution exceeding 80%, and a V-0 flame-retardant rating. Compared with the representative polymer functional composites involved, the FR PLA-based composite prepared in this work mainly exhibits the advantages of good flame retardancy, EMI shielding performance, FDM processability, and relatively low conductive filler loading, as indicated in the Supplementary Material in Table S1 [11,16,20,21,52].

**Figure 10:**
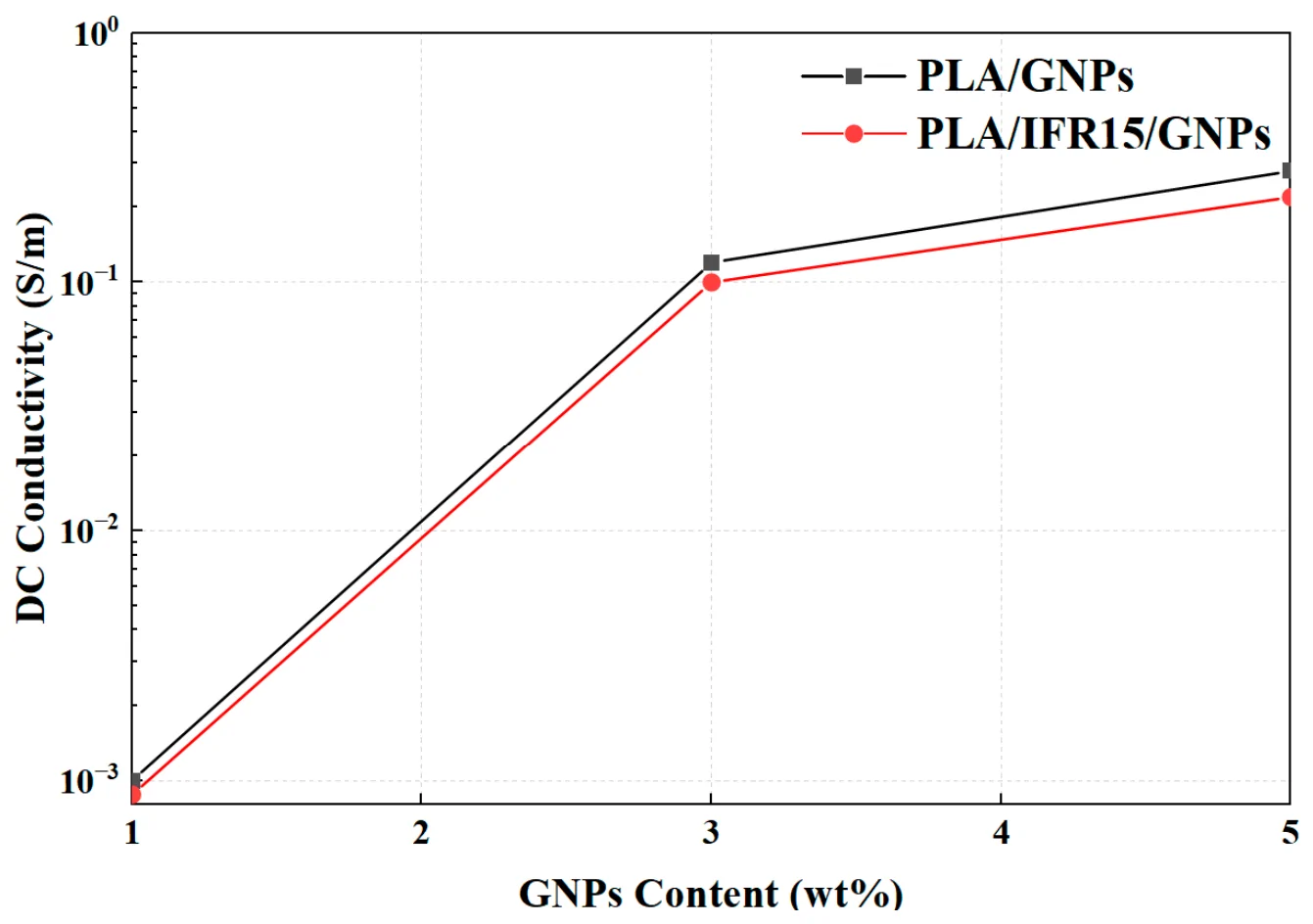
Electrical conductivity as a function of GNPs content.

The direct-current electrical conductivity (σ) of the composites was measured, and Figure 10 shows the variation in electrical conductivity as a function of GNPs content. Neat PLA is an electrical insulator. Upon the addition of 1 wt% GNPs, the electrical conductivity increased sharply to 10^−3^ S/m. When the GNPs content reached 3 wt%, the conductivity further rose to 1.2 × 10^−1^ S/m. With a further increase to 5 wt%, the conductivity climbed to 0.28 S/m, yet with a noticeably slower growth rate. This marked change in conductivity indicates a possible percolation transition associated with the progressive formation of conductive GNPs networks in the PLA matrix, which need further investigation. At very low filler contents, GNPs are mainly dispersed as isolated or weakly connected nanosheets. As the GNPs content increases, the interparticle distance gradually decreases, and adjacent nanosheets begin to contact and overlap, leading to the formation of a continuous conductive network. The obvious increase in conductivity suggests that the conductive network develops rapidly in the lower loading region (up to 3 wt% GNPs). With the further addition of GNPs, the network becomes increasingly established, and the increase in conductivity therefore becomes more gradual [16]. After the incorporation of 15 wt% IFR, the electrical conductivity at the same GNPs content decreases slightly, owing to the insulating nature of IFR diluting the conductive pathways. Nevertheless, the overall conductivity is sufficiently high to support effective EMI shielding. Accordingly, PLA/IFR15/GNPs3 exhibited an SE_T_ of 20.6 dB at a conductivity of 0.098 S/m. A moderate conductivity may also help prevent excessive interface reflection, thereby favoring an absorption-dominated shielding behavior.

**Figure 11:**
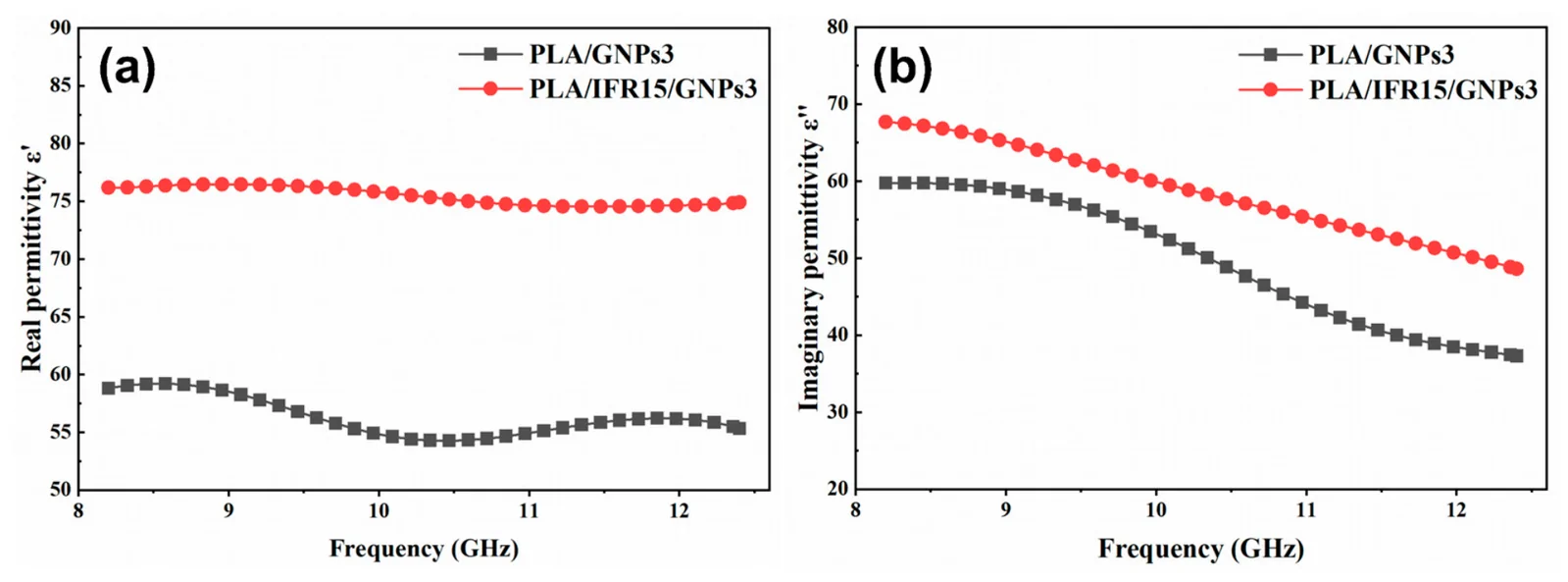
Frequency dependence of (**a**) the real permittivity ε’ and (**b**) the imaginary permittivity ε” of PLA/GNPs3 and PLA/IFR15/GNPs3 in the X-band.

The correlated dielectric loss of the composites was also investigated, and the complex permittivity of PLA/GNPs3 and PLA/IFR15/GNPs3 was evaluated in the X-band, as shown in Figure 11. The real permittivity ε’ of PLA/IFR15/GNPs3 remained substantially higher than that of PLA/GNPs3 across the entire frequency range. Meanwhile, the imaginary permittivity ε”, which reflects dielectric energy dissipation, also shows a consistently higher value for PLA/IFR15/GNPs3 than for PLA/GNPs3. Both composites exhibited frequency-dependent dielectric responses, with ε’’ gradually decreasing with increasing frequency. Notably, despite a slight decrease in electrical conductivity upon IFR incorporation, the imaginary permittivity (ε”) increases markedly. This observation indicates that the enhanced dielectric loss cannot be solely attributed to conduction loss. Instead, the introduction of heterogeneous interfaces among PLA, IFR, and GNPs could promote interfacial polarization and charge accumulation under the alternating electromagnetic field, leading to enhanced polarization-related dielectric loss. This additional loss mechanism contributes substantially to electromagnetic wave attenuation and is consistent with the absorption-dominated shielding behavior observed in this system.

### 3.6. Construction of Structural Model for Flame-Retardant/EMI Shielding Synergy

**Figure 12:**
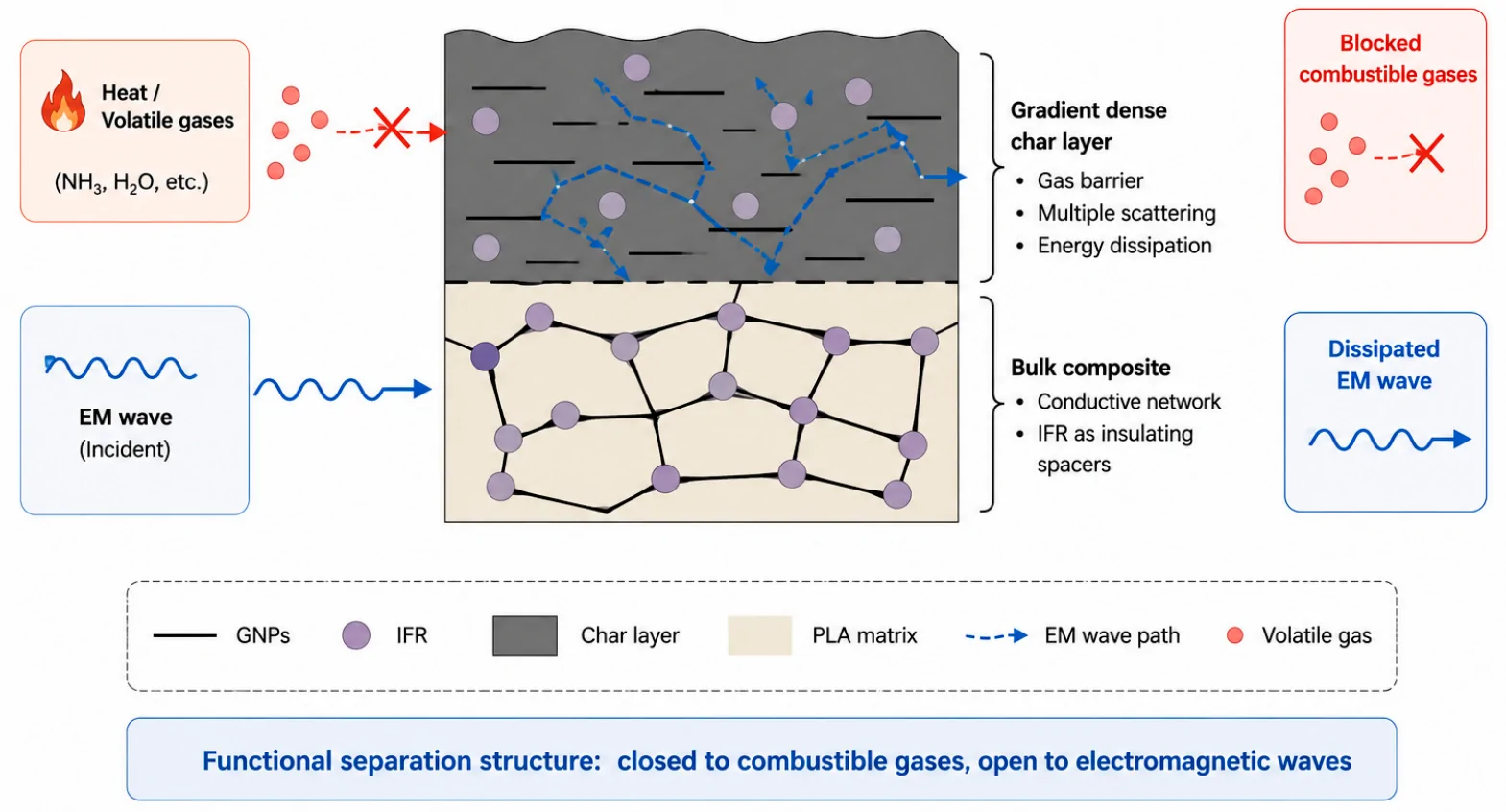
Schematic illustration of the synergistic flame-retardant and electromagnetic interference shielding model.

Based on the above analyses, a synergistic structural model for flame retardancy and EMI shielding is proposed, as illustrated in Figure 12. In the PLA/IFR15/GNPs3 system, the material exhibits a “selective regulation” behavior toward different energy carriers, including heat, flammable volatiles, and electromagnetic waves. GNPs construct a percolated conductive network within the system, providing efficient pathways for electromagnetic wave transport and dissipation, where conduction loss and interfacial polarization loss are the dominant mechanisms. The incorporation of IFR introduces discrete insulating domains within this conductive network, disrupting its excessive continuity. Such disruption suppresses excessive reflection and improves impedance matching, thereby driving a transition from a reflection-dominated to an absorption-dominated shielding mechanism. Meanwhile, upon thermal stimulation, IFR preferentially decomposes to release gases and generate phosphorus-rich species, promoting the formation of a gradient-dense char layer. This char layer acts as an effective barrier against the release of flammable volatile products during combustion. Meanwhile, the conductive and heterogeneous structure of the char layer may provide additional pathways for electromagnetic wave attenuation. Furthermore, GNPs are embedded into the char framework, reinforcing its structural integrity and prolonging the propagation path of electromagnetic waves within the material. Consequently, the system essentially establishes a functional separation architecture characterized by blocking flammable gases while dissipating electromagnetic waves, achieving coordinated enhancement of both flame retardancy and absorption-dominated EMI shielding performance.

## 4. Conclusions

In this study, FR PLA-based composites with good flame-retardant and electromagnetic interference (EMI) shielding properties were fabricated via melt compounding combined with fused deposition modeling (FDM) 3D printing. Microstructural observations showed that the formulation containing 15 wt% IFR and 3 wt% GNPs exhibited the most favorable filler distribution and interfacial morphology among the investigated compositions. At 700 °C, PLA/IFR15/GNPs3 retained 14.3% char residue, compared with only 0.8% for neat PLA. In terms of flame retardancy, PLA/IFR15/GNPs3 exhibited a limiting oxygen index (LOI) of 32.5% and attained a UL-94 V-0 rating. Cone calorimeter results further demonstrated that the peak heat release rate (pHRR) and total heat release (THR) of the composite decreased by 29.4% and 47.3% compared with neat PLA, accompanied by the formation of a denser and more continuous char layer with fewer cracks, which corroborates the synergistic flame-retardant mechanism of IFR and GNPs in both the gas and condensed phases. Regarding EMI shielding performance, the composite delivered a total shielding effectiveness (SE_T_) of 20.6 dB in the X-band (8.2–12.4 GHz) with an absorption contribution exceeding 80%, indicating an absorption-dominated shielding mechanism. Collectively, this work demonstrates that the synergistic design of IFR and GNPs, combined with FDM 3D printing, offers a viable route to multifunctional PLA composites that integrate flame retardancy and EMI shielding, providing both fundamental insights and practical guidance for the development of high-performance multifunctional 3D-printed polymer materials.

## Figures and Tables

**Table 1:** Composition of FR PLA-based composite samples.

Sample Number	PLA (wt%)	IFR (wt%)	GNPs (wt%)
PLA	100	0	0
PLA/IFR10	90	10	0
PLA/IFR15	85	15	0
PLA/IFR20	80	20	0
PLA/GNPs1	99	0	1
PLA/GNPs3	97	0	3
PLA/GNPs5	95	0	5
PLA/IFR15/GNPs1	84	15	1
PLA/IFR15/GNPs3	82	15	3
PLA/IFR15/GNPs5	80	15	5

**Table 2:** FDM 3D printing parameters used for sample fabrication.

Printer Model	Nozzle Diameter (mm)	Layer Height (mm)	Infill Pattern	Infill Density (%)	Bed Temperature (°C)	Nozzle Temperature (°C)
CreatBot F430 Nx	0.4	0.2	−60°\0°\+60°	100	60	210

**Table 3:** Evaluation of printing performance of filaments with different GNPs contents.

Sample	GNPs (wt%)	Extrusion Stability	Filament Diameter Fluctuation	Surface Quality	Printability
PLA	0	Excellent	±0.02 mm	Smooth	Printable
PLA/IFR15	0	Excellent	±0.02 mm	Smooth	Printable
PLA/IFR15/GNPs1	1.0	Good	±0.03 mm	Smooth	Printable
PLA/IFR15/GNPs3	3.0	Good	±0.03 mm	Slightly rough	Printable
PLA/IFR15/GNPs5	5.0	Poor	±0.08 mm	Rough with particles	Difficult to print

**Table 4:** The interlayer shear strength of printed specimens.

Sample	GNPs (wt%)	Interlayer Shear Strength (MPa)
PLA/IFR15	0	28.5 ± 1.2
PLA/IFR15/GNPs1	1.0	29.2 ± 1.0
PLA/IFR15/GNPs3	3.0	31.5 ± 0.9
PLA/IFR15/GNPs5	5.0	30.1 ± 1.1

**Table 5:** Characteristic TGA parameters of composites (nitrogen atmosphere, 10 °C/min heating rate).

Sample Number	T_5%_ (°C)	T_max_ (°C)	Char Yield at 700 °C (%)
PLA	329.5 ± 1.2	361.8 ± 0.8	0.8 ± 0.2
PLA/IFR15	305.2 ± 1.5	348.5 ± 1.1	9.6 ± 0.5
PLA/GNPs3	324.8 ± 1.0	358.2 ± 0.9	4.1 ± 0.3
PLA/IFR15/GNPs3	298.7 ± 1.8	340.2 ± 1.3	14.3 ± 0.7

**Table 6:** LOI and UL-94 test results of different composite materials.

Sample	Afterflame Time t_1_ + t_2_ (s)	Dripping Behavior	Cotton Ignition	UL-94 Rating	LOI (%)
PLA	>30	Y	Y	NR	20.5 ± 0.3
PLA/IFR10	14 ± 1	Y	N	V-1	23.2 ± 0.4
PLA/IFR15	12 ± 2	Y	N	V-1	27.8 ± 0.5
PLA/IFR20	11 ± 1	Y	N	V-1	28.1 ± 0.4
PLA/GNPs1	>30	Y	Y	NR	20.9 ± 0.2
PLA/GNPs3	>30	Y	Y	NR	21.5 ± 0.3
PLA/GNPs5	>30	Y	Y	NR	22.0 ± 0.4
PLA/IFR15/GNPs1	8 ± 1	N	N	V-0	29.2 ± 0.5
PLA/IFR15/GNPs3	3 ± 1	N	N	V-0	32.5 ± 0.6
PLA/IFR15/GNPs5	5 ± 1	N	N	V-0	31.6 ± 0.5

**Table 7:** Key parameters from cone calorimeter tests.

Sample	TTI (s)	pHRR (kW/m^2^)	THR (MJ/m^2^)	Average Mass Loss Rate (g/s)	pSPR (m^2^/s)	TSP (m^2^)	Residual Char Yield (%)
Neat PLA	57 ± 2	336.8 ± 15	52.0 ± 3.5	0.052 ± 0.003	0.038	1.4	4.8
PLA/IFR15	43.4 ± 3	270.4 ± 13	36.4 ± 2.8	0.041 ± 0.002	0.058	4.4	19.4
PLA/GNPs3	53 ± 2	313.3 ± 11	46.4 ± 3.1	0.048 ± 0.002	0.022	1.1	13.2
PLA/IFR15/GNPs3	55 ± 1	237.6 ± 18	27.4 ± 2.2	0.032 ± 0.001	0.063	3	25.7

**Table 8:** Average electromagnetic shielding parameters of composites in the 8.2–12.4 GHz range (sample thickness: 2.0 mm).

Sample	SE_T_ (dB)	SE_A_ (dB)	SE_R_ (dB)	Absorption Contribution (%)
PLA	0.8 ± 0.2	0.5	0.3	62.5
PLA/GNPs1	6.6 ± 0.5	4.2	2.4	63.6
PLA/GNPs3	21.4 ± 0.7	17.4	4.0	81.3
PLA/GNPs5	25.9 ± 1.2	21.5	4.4	83.0
PLA/IFR15/GNPs1	5.6 ± 0.7	3.5	2.1	62.5
PLA/IFR15/GNPs3	20.6 ± 1.0	16.9	3.7	82.0
PLA/IFR15/GNPs5	18.3 ± 1.2	15.0	3.4	81.9

## Data Availability

The original contributions presented in this study are included in the article/Supplementary Material. Further inquiries can be directed to the corresponding authors.

## Supplementary Materials

The following supporting information can be downloaded at: https://www.mdpi.com/article/10.3390/polym18182221/s1, Table S1. The flame-retardancy and EMI shielding performances of representative FR polymer composites presented in literatures and this work
