# Supplementary material for: FDM 3D Printing of Flame-Retardant Polylactic-Acid-Based Composites with Electromagnetic Interference Shielding Performance
Source: Polymers (Basel). 2026 Sep 12;18(18):2221. doi: 10.3390/polym18182221 (PMC13611730; doi:10.3390/polym18182221)
Supplement: Supplementary file 1 [file polymers-18-02221-s001.zip › polymers-4462891-supplementary.pdf]

FDM 3D Printing of Flame-Retardant Polylactic-Acid-Based Composites with Electromagnetic Interference Shielding Performance

Jun Li [1], Zijie Xu [1], Qinniu Lv [1], Xin Wang [2*], Yinghong Chen [1*]

[1] State Key Laboratory of Advanced Polymer Materials, Polymer Research Institute of Sichuan University, Chengdu, 610065, China; lj03040010@163.com (J.L.); xuzijie0310@163.com (Z.X.); lvqinniu_scu@163.com (Q.L.)

[2] Research Center for Composite Materials, State Key Laboratory of Advanced Refractories, Shanghai University, Shanghai 200444, China

[*] Correspondence: xin_wang@shu.edu.cn (X. Wang), johnchen@scu.edu.cn (Y. Chen)

1. Determining composition of FR PLA-based Composites

The design of formulations presented in Table 1 is mainly based on the consideration of balance between the cost-effectiveness and the performance. Here, the intumescent flame retardant (IFR) used in this paper consists of ammonium polyphosphate (APP) and pentaerythritol (PER), and the weight ratio of APP and PER is fixed at APP:PER=3:1. We determined the IFR loading at a lower level (in the range of 10-20 wt%) mainly considering that the added IFRs should not negatively influence the mechanical performance of PLA material much while keeping a relatively satisfactory flame retardancy (at least not lower than UL94 V-1 rating). This also lays a foundation for further improving the flame retardancy of PLA and imparting PLA the electromagnetic interference (EMI) shielding property through adding other functional fillers such as graphene (GNP). It is found that with increasing IFR content from 10 to 20 wt%, the corresponding flame retardancy exhibits a first obvious and then slow increase (LOI: 23.2% (10 wt% IFR) → 27.8% (15 wt% IFR) → 28.1% (20 wt%)) and the turning point is at 15 wt%. So, the IFR loading is fixed at 15 wt% for further study. In order to prepare the FR EMI PLA material, the conductive functional GNP fillers were additionally incorporated. The low loading of GNP (1–5 wt%) was chosen by just referring to our previous studies on FDM-printed PLA/graphene composites [1,3] and also considering the high cost of GNP and easy agglomeration of GNP at higher loading.

2. Comparison with FR polymer composites reported in literatures

The representative flame-retarded/EMI-shielded polymer composites reported in literatures were compared with the PLA/IFR15/GNPs3 functional composite prepared in this work (Table S1). As can be seen, although there are the functional composite systems which exhibit the relatively higher EMI shielding effectiveness, they have the higher conductive filler contents, complex porous architecture construction, additional surface-modification processes, and absence of integrating flame retardancy and EMI shielding property. In contrast, the PLA/IFR15/GNPs3 composite developed in this paper could simultaneously achieve flame retardancy of LOI 32.5% and UL-94 V-0 rating, and EMI shielding effectiveness of 20.6 dB using only 3 wt% loading of GNPs through a cost-effective melt-compounding–filament-extrusion–FDM route, reaching a favorable balance among flame retardancy, EMI shielding performance, relatively low conductive filler loading, and FDM processability rather than in solely optimizing a single performance parameter.

Table S1. The flame-retardancy and EMI shielding performances of representative FR polymer composites presented in literatures and this work

System	Matrix/filler	Preparation method	Filler loading	Flame retardancy	EMI SE	Reference
PLA/GNP-coated porous lattice	PLA/GNP	FDM + surface coating	5.56 wt% GNP	LOI = 27.5%	~25 dB	[2]
PLA/GNs	PLA/GNs	melt blending + FDM	9.08 vol% GNs	Not reported	34.9 dB	[3]
Graphene/PU	PU/graphene	foaming	1.0 wt% graphene	Reduced pHRR	~30 dB	[4]
PLA fabric/MXene	PLA/MXene	surface modification	PA-PEI-MXene -60	LOI = 35.6%	54 dB	[5]
PLA/PCL/10APP /8CNT/1.61IPU	PLA/PCL/APP/ CNT	melt blending	10 wt% APP + 8 wt% CNT + 1.6 wt% IPU	LOI = 30.3%; V-0	33.9 dB	[6]
PLA/IFR15/GNPs3	PLA/IFR/GNPs	melt blending + FDM	15 wt% IFR + 3 wt% GNPs	LOI = 32.5%; V-0	20.6 dB	This work

References

[1] Shi, S.; Peng, Z.; Jing, J.; Yang, L.; Chen, Y.; Kotsilkova, R.; Ivanov, E. Preparation of highly efficient electromagnetic interference shielding polylactic acid/graphene nanocomposites for fused deposition modeling three-dimensional printing. *Ind. Eng. Chem. Res.* **2020**, *59*, 15565–15575. https://dx.doi.org/10.1021/acs.iecr.0c02400

[2] Taala, N.; Aberoumand, M.; Ribeiro dos Anjos, E. G.; Sundararaj, U. Surface-percolated GNP coatings on 3D-printed PLA lattices for tunable EMI shielding and flame-retardant multifunctionality. *Nanoscale* **2026**, *18*, 8813–8836. https://doi.org/10.1039/d5nr05018a

[3] Shi, S.; Dai, M.; Tao, X.; Wu, F.; Sun, J.; Chen, Y. 3D printed polylactic acid/graphene nanocomposites with tailored multifunctionality towards superior thermal management and high-efficient electromagnetic interference shielding. *Chem. Eng. J.* **2022**, *450,* 138248. https://doi.org/10.1016/j.cej.2022.138248

[4] Yao, Y.; Jin, S.; Ma, X.; Yu, R.; Zou, H.; Wang, H.; Lv, X.; Shu, Q. Graphene-containing flexible polyurethane porous composites with improved electromagnetic shielding and flame retardancy. *Compos. Sci. Technol.* **2020**, *200,* 108457. https://doi.org/10.1016/j.compscitech.2020.108457

[5] Chu, C.; Gao, Y.; Ma, X.; Cheng, R.; Xu, P.; Ding, Y. Multilevel structural polylactic acid fabrics for flame retardancy, durability, and electromagnetic interference shielding. *Int. J. Biol. Macromol.* **2024**, 282, 136934. https://doi.org/10.1016/j.ijbiomac.2024.136934

[6] Wang, Z.; Yan, T.; Gao, Y.; Ma, X.; Xu, P.; Ding, Y. Improving flame retardant and electromagnetic interference shielding properties of poly(lactic acid)/poly(ε-caprolactone) composites using catalytic imidazolium modified CNTs and ammonium polyphosphate. *Int. J. Biol. Macromol.* **2024**, *259*, 129265. https://doi.org/10.1016/j.ijbiomac.2024.129265
